# Association between subjective well-being and all-cause mortality among older adults in China

**DOI:** 10.1186/s12888-023-05079-y

**Published:** 2023-08-25

**Authors:** Chunsu Zhu, Zhiwei Lian, Yongying Huang, Qiaofeng Zhong, Jianmin Wang

**Affiliations:** 1 Clinical Oncology School of Fujian Medical University, Fujian Cancer Hospital, No. 420, Fuma Road, Jinan District, Fuzhou, 350014 China; 2 Fujian Key Laboratory of Advanced Technology for Cancer Screening and Early Diagnosis, Clinical Oncology School of Fujian Medical University, Fujian Cancer Hospital, Fuzhou, China

**Keywords:** Subjective well-being, All-cause mortality, Cohort study

## Abstract

**Background:**

Although several studies in high-income countries have suggested a positive association between subjective well-being (SWB) and mortality, studies conducted in low- and middle-income countries, such as China, are scarce. The purpose of this study is to examine the association between SWB and all-cause mortality among the older Chinese population.

**Methods:**

Data were from the Chinese Longitudinal Healthy Longevity Survey (CLHLS), a population-based longitudinal cohort study in 22 of 31 provinces in mainland China. A total of 13,282 individuals aged 65 ≥ years who were recruited in 2002 and followed-up until 2018 were included. SWB was assessed with an eight-item tool covering life satisfaction, positive affect (including optimism, happiness, personal control and conscientiousness) and negative affect (including anxiety, loneliness and uselessness). Cox proportional hazards regression methods were carried out to estimate the association between SWB and total mortality, adjusting for a wide range of potential confounders. Subgroup analyses and interaction analyses were further conducted.

**Results:**

During the 16.5 years of follow-up, 8459 deaths were identified. Greater SWB was independently associated with a reduced risk of all-cause mortality (adjusted hazard ratio [HR] = 0.85, 95% confidence interval [CI] = 0.81–0.89) after adjustment for age, sex, marital status, education level, place of residence, smoking status, drinking, exercise, diet, BMI, hypertension, diabetes, heart disease, cerebrovascular diseases and cancer. Of the eight individual SWB symptoms, only 2 items, feelings of uselessness (adjusted HR = 0.94, 95% CI = 0.89–0.99) and happiness (adjusted HR = 0.91, 95% CI = 0.86–0.95), were significantly associated with total mortality. Associations remained significant across all subgroups regardless of different characteristics.

**Conclusions:**

Higher SWB overall and 2 certain symptoms (feelings of uselessness and happiness) were independently associated with all-cause mortality risk among older Chinese adults. The association was consistent across different groups, suggesting that promoting a healthier SWB may be beneficial to all older individuals irrespective of their characteristics.

**Supplementary Information:**

The online version contains supplementary material available at 10.1186/s12888-023-05079-y.

## Introduction

Population aging, a primary contributor to a lower quality of life in elderly individuals, has become a global challenge in eldercare, especially in low- and middle-income countries (LMICs) [1], such as China. China is one of the major LMICs and is experiencing rapid population aging. It was estimated that China houses approximately 18% of the world’s population, with 164.5 million citizens aged 65 years and above, and 26 million aged 80 years and above in 2019 [2]. Subjective well-being (SWB) is an important quality of life indicator and has gained growing interest in recent decades [3]. SWB is commonly defined as ones’ subjective evaluations of their lives [4], which mainly contain three components: judgments about their life satisfaction, positive affect (e.g., happiness, optimism) and negative affect (e.g., anxiety, loneliness) [5]. At older ages, restrictions in mobility [6], declines in physical health status and cognitive functions [7], and a lack of social support from family members may result in negative perceptions of aging [8, 9]. Consequently, older people are more likely to have lower SWB than their counterparts [8, 10]. SWB may increase the risk of mortality through several pathways including healthy lifestyles (e.g., physical activities and dietary choices) and biological mechanisms (e.g., inflammatory, neuroendocrine, and metabolic pathways) [11].

Previous studies have suggested a positive association between greater SWB levels and reduced mortality risk [12–14]. However, the evidence is inconsistent and incomparable [15] due to the broad variety of SWB measures (e.g., the majority of existing studies focus on only one aspect of SWB, such as happiness or optimism) [16] and differences in study methodology (e.g., adjusting confounders). A recent meta-analysis reported that SWB acts as a protective factor for mortality with a pooled hazard ratio of 0.92 [17], but a high level of heterogeneity and publication bias may restrict the generalizability of its findings. In addition, the studies included in this meta-analysis were mainly conducted in developed countries (e.g., US and Europe), which may not be properly applied to LMICs because of cultural differences. For instance, the survival of older people is more selective in LMICs, and societies may have different pressures and expectations on their aging population [18, 19]. A few studies have assessed the relationship between SWB and mortality in Chinese people, but they are limited either by small sample sizes or by focusing on one region of China (e.g., rural areas or Taiwan) [20, 21]. For example, a recent study of rural elderly people reported that individuals with SWB equal to the average level experienced a 10% reduction in the risk of death [21]. And a survey of 5131 adults in Taiwan also revealed a protective effect of life satisfaction on the risk of mortality [20]. Nevertheless, large-scale studies investigating the association between SWB and mortality in older Chinese people are still needed. In addition, to date, no investigator has taken the contribution of individual SWB symptoms to mortality into consideration in one study simultaneously, which also gives us an impetus to assess the relationships between individual SWB symptoms and mortality. Since both SWB level and mortality risk are different across participants with diverse characteristics (e.g., sex, age), the relationship between SWB and total mortality risk may differ among different subgroups. However, this remains less understood.

Therefore, the purpose of the present study is threefold. First, we assessed the association between SWB and the risk of total mortality within a nationally representative cohort study of older Chinese individuals. Second, we examined the associations between specific SWB symptoms and total mortality. Third, we further explored whether these associations are consistent in subgroups with different characteristics.

## Methods

### Study population

Data used in this study were taken from the Chinese Longitudinal Healthy Longevity Survey (CLHLS), a nationwide prospective cohort study among community-dwelling older people in China. A detailed description of the CLHLS project can be found elsewhere [22]. In brief, beginning in 1998, subsequent follow-up surveys of the CLHLS were carried out every 2–3 years, with a response rate of approximately 90% for each wave. To ensure representativeness, a multilevel cluster sampling method was adopted in the project. In total, 631 cities or countries were randomly selected from 22 to 31 provinces in China, covering 85% of China’s total population. Information on demographic characteristics, lifestyles and health status was collected by well-trained and qualified interviewers with standardized questionnaires at the interviewers’ homes.

In the current study, data from the 2002, 2005, 2008, 2011, 2014, and 2018 waves of the CLHLS were used. The first wave of the CLHLS (1998) was not included in this analysis because the 1998 wave did not include individuals aged 65 to 79 years. In total, 16,064 participants were surveyed in 2002. We excluded participants who were unable to answer SWB items (n = 2303, 14.3%) and those with missing values for lifestyles (n = 382, 2.4%), chronic physical comorbidities (n = 12, 0.07%) and demographic characteristics (n = 85, 0.53%). We did not impute the missing data because less than 5% of the data were missing. Finally, 13,282 participants were included in the analysis.

### Assessment of subjective well-being

SWB was measured by a previously developed tool based on a five-point Likert scale [23], which included eight items covering life satisfaction, positive emotions (optimism, happiness, personal control and conscientiousness) and negative emotions (anxiety, loneliness, and uselessness). Life satisfaction was assessed with the question “How do you rate your life at present?”, ranging from 1 (very bad) to 5 (very good). Questions “do you always look on the bright side of things?” “are you as happy as when you were younger?” “can you make your own decisions concerning your personal affairs?” “do you like to keep your belongings neat and clean?” “do you often feel fearful or anxious?” “do you feel lonely and isolated?” and “do you feel the older you get, the more useless you are?” were used to measure the affective aspects for participants. The answers for each item were coded from 1 (never) to 5 (always), and negative items (including feelings of anxiety, loneliness, and uselessness) were reversely coded. The total SWB score was calculated by summing scores across all items, contributing to a maximum SWB score of 40, with higher scores indicating more positive SWB. The SWB score was split into quartiles (Q1 = 0–25, Q2 = 26–27, Q3 = 28–30, Q4 = 31–40), following previous research [23, 24]. Participants who had an SWB score within the range of Q4 (SWB score ≥ 31) were regarded as having a better SWB status, whereas those who had an SWB score lower than 31 were classified as having a worse SWB status [23]. The Cronbach’s α for internal consistency was 0.68 [23], and the confirmatory factor analysis in a previous study suggested that the unidimensional construct of this SWB scale has sufficient validity to assess SWB in older people [25].

### Assessment of mortality

The survival status and date of death were collected in the follow-up waves in 2005, 2008, 2011, 2014 and 2018. The dates of death were collected based on the officially issued death certificate when available, or otherwise through interviews with close family members or village doctors. The mortality data in the CLHLS were shown to be of high quality in previous studies [26, 27]. The participants who survived in the 2018 wave or who were lost to follow-up were regarded as censored. The person years were calculated from 2002 (baseline) to the dates of death, loss to follow-up (3943 of 16,064 [24.5%]) or the date of last interview in 2018, whichever came first.

### Covariates

Covariates at baseline were collected by trained interviewers with a standardized questionnaire. Three types of covariates were included following previous studies [14, 28]: demographic characteristics, lifestyle-related factors, and chronic physical comorbidities. The demographic characteristics included age, sex, marital status (married, divorced/widowed/separated/singled), educational level (illiteracy, literacy), and place of residence (city/urban, rural). Lifestyle-related factors included smoking status (never, current/former smoker), drinking status (never, current/former drinker), regular exercise (yes, no), dietary diversity (good, poor), and body mass index (BMI, normal weight [18.5 ≤ BMI < 24.0 kg/m^2^], underweight [BMI < 18.5 kg/m^2^] or overweight/obese [BMI ≥ 24.0 kg/m^2^]) [29, 30]. The question “ Do you do exercise regularly at present, including jogging, playing ball, running or Qigong?”, and the answers were recorded as yes or no [27]. At baseline, a food frequency questionnaire was used to collect food consumption data, and seven major food groups were surveyed, including fruits, vegetables, meat, fish, eggs, beans and tea. A total dietary diversity score was calculated by summing scores across all items, with a maximum score of 7. Participants who had a dietary diversity score at or over the average value were regarded as having good dietary diversity [31, 32]. Chronic physical comorbidities dealt with self-reported doctors’ diagnosis of noncommunicable diseases, including hypertension, diabetes, cerebrovascular diseases, cancer and heart disease.

### Statistical analysis

All analyses were conducted in the Statistical Package for the Social Sciences (SPSS), version 21.0 and R software 3.6.1. A two-sided p < 0.05 was regarded as statistically significant. Descriptive statistics of baseline characteristics are presented by SWB status, continuous variables are presented as the means and standard deviations (SDs), and categorical variables are shown as frequencies with percentages. T tests and chi-square tests were used to compare the differences between groups, as appropriate.

To estimate the association between SWB and all-cause mortality, Cox proportional hazards regression models were used to calculate the hazard ratios (HRs) and 95% confidence intervals (CIs). Three models were fitted: model 1 was adjusted for age and sex; model 2 was adjusted for age, sex, marital status, educational level, place of residence, smoking, drinking, dietary diversity, exercise and BMI; and model 3 was further adjusted for history of hypertension, diabetes, cerebrovascular diseases, cancer and heart disease. The Wald test was performed to evaluate the linear trends, with the total SWB score quartile as a continuous variable. The proportional hazards assumption was verified graphically, and there was no evidence of obvious departure from the assumption. To assess the association between specific SWB symptoms and all-cause mortality, we coded each item as a dichotomous variable by defining the responses of always or often as having symptoms using the method of previous studies [33]. All eight items were entered simultaneously in model 3, and negative items were coded reversely.

Stratification analyses by all aforementioned covariates were conducted to explore whether the potential relationships between SWB and all-cause mortality were moderated by these factors. P values for interactions were evaluated for all covariates using interaction terms. To reduce the false discovery rate, a lower p value was used for subgroup and interaction analyses (two-tailed p value < 0.01). To test the robustness of the study, several sensitivity analyses were conducted. First, all the analyses were repeated in the following scenarios: (1) excluding those aged 90 years and above at baseline (n = 4926); (2) excluding participants who died within the first year of follow-up (n = 2752); (3) excluding individuals who were underweight (BMI < 18.5 kg/m^2^, n = 119); and (4) excluding those with life-threatening diseases at baseline (including cancer, heart disease and cerebrovascular diseases n = 2991). Second, approximately 14.3% of the participants selected “unable to answer” for the SWB items, and it was estimated that approximately 90% of these responses were attributable to poor health [34], which might affect their mortality risk. Therefore, we classified those participants into a separate group and compared the mortality risk among groups.

## Results

Of the 13,282 included participants, the mean (SD) age at baseline was 84.7 (11.5) years; 5929 (44.6%) were male, and 7353 (55.4%) were female. The characteristics of the participants were shown in Table 1. At baseline, 5790 participants (43.6%) had better SWB (SWB score ≥ 31). Compared with those with worse SWB, participants with better SWB were more likely to be younger, be male, live in an urban or city setting, be married, be literate, take regular exercise, have good dietary diversity and have normal weight (all p < 0.001).

**Table 1:** Baseline characteristics stratified by subjective well-being status

Characteristics	Participants, No. (%)			P value
	Total sample (N = 13,282)	Subjective well-being		
		Worse(n = 7492)	Better(n = 5790)	
Age, mean(SD), y	84.7(11.5)	85.8(11.4)	83.4(11.5)	< 0.001
Men	5929(44.6)	2993(39.9)	2936(50.7)	< 0.001
Urban/city residence	6171(46.5)	2991(39.9)	3180(54.9)	< 0.001
Married	4379(33.0)	2053(27.4)	2326(40.2)	< 0.001
Education level				< 0.001
Illiteracy	7879(59.3)	4960(66.2)	2919(50.4)	
literacy	5403(40.7)	2532(33.8)	2871(49.6)	
Smoking status				< 0.001
Current/former	4797(36.1)	2419(32.3)	2378(41.1)	
Never	8485(63.9)	5073(67.7)	3412(58.9)	
Drinking status				
Current/former	4767(35.9)	2482(33.1)	2285(39.5)	< 0.001
Never	8515(64.1)	5010(66.9)	3505(60.5)	
Regular exercise				< 0.001
No	8685(65.4)	5658(75.5)	3027(52.3)	
Yes	4597(34.6)	1834(24.5)	2763(47.7)	
Diet diversity				< 0.001
Poor	5747(43.3)	3956(52.8)	1791(30.9)	
Good	7535(56.7)	3536(47.2)	3999(69.1)	
BMI, kg/m2				< 0.001
Underweight/overweight/obese	7363(55.4)	4467(59.6)	2896(50.0)	
Normal weight	5919(44.6)	3025(40.4)	2894(50.0)	
History of comorbidities				
Hypertension	3468(26.1)	1881(25.1)	1587(27.4)	0.003
Diabetes	1777(13.4)	908(12.1)	869(15.0)	< 0.001
Cerebrovascular disease	1860(14.0)	991(13.2)	869(15.0)	0.003
Cancer	1206(9.1)	581(7.8)	625(10.8)	< 0.001
Heart disease	2501(18.8)	1281(17.1)	1220(21.1)	< 0.001

SD: standard deviation; BMI: body mass index

**Fig. 1:**
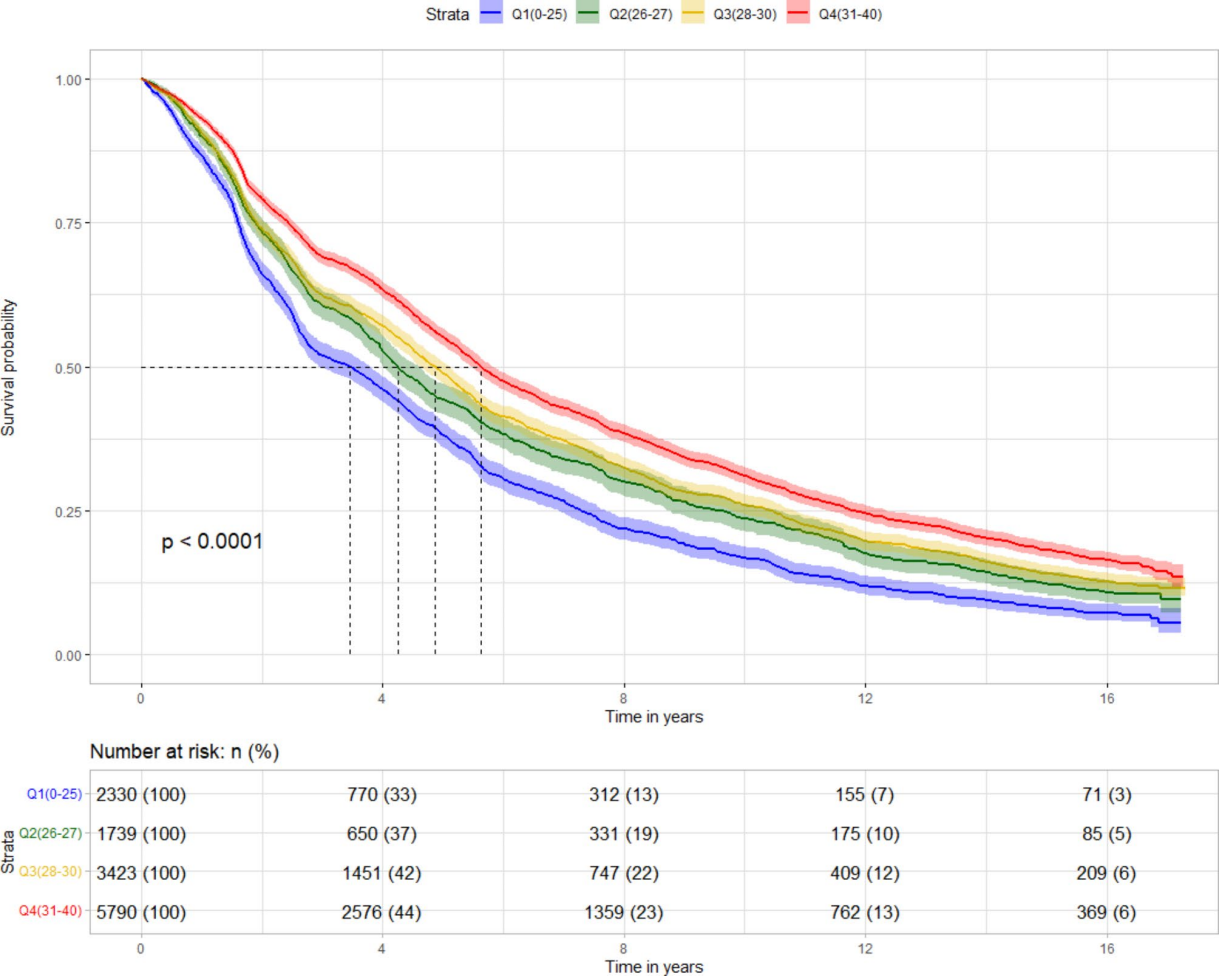
Survival of older individuals with different level of subjective well-being. The log-rank test was used. Shade was used to show the 95% CI for the association between subjective well-being and all-cause mortality

During the follow-up period between 2002 and 2018, 8459 deaths (64.4%) were identified. Table 2 showed the association between SWB and all-cause mortality. After adjusting for all potential confounders (model 3), the participants with better SWB at baseline experienced a 15% lower risk of death than those with worse SWB (adjusted HR = 0.85, 95% CI: 0.81–0.89). Similar results were found when the total SWB score was modeled as quartiles (Table 2). After adjusting for confounding factors, in comparison to quartile 1 (Q1), the adjusted HR for Q4 was 0.77 (95% CI: 0.73–0.82), and a linear trend between SWB quartiles and all-cause mortality was also found using the Wald test (p < 0.001) (Table 2). Figure 1 shows that there was a higher survival in the participants with a better SWB status, and lower survival in the participants with a worse SWB score (p < 0.001).

**Table 2:** Associations between subjective well-being and all-cause mortality, n = 13, 282

Variables	Death, No.	Participants No.	HR (95%CI)		
			Model 1	Model 2	Model 3
SWB					
Worse	5103	7492	1 (Ref)	1 (Ref)	1(Ref)
Better	3446	5790	0.82 (0.79,0.86)	0.85 (0.81,0.89)	0.85 (0.81,0.89)
SWB score, continuous	8549	13,282	0.97(0.97,0.98)	0.98 (0.97,0.98)	0.98 (0.97,0.98)
SWB score, quartile					
Q1(0–25)	1646	2330	1 (Ref)	1 (Ref)	1 (Ref)
Q2(26–27)	1163	1739	0.90 (0.83,0.97)	0.90 (0.84,0.98)	0.91 (0.84,0.98)
Q3(28–30)	2294	3423	0.85 (0.79,0.90)	0.86 (0.81,0.92)	0.87 (0.81,0.92)
Q4(31–40)	3446	5790	0.74 (0.70,0.79)	0.77 (0.72,0.82)	0.77 (0.73,0.82)
P trend			< 0.001	< 0.001	< 0.001

HR: hazard ratio; CI: confidence interval; Ref: reference; SWB: subjective well-being. Model 1 was adjusted for age and sex; model 2 was adjusted for age, sex, marital status, educational level, place of residence, smoking, drinking, dietary diversity, exercise and BMI; and model 3 was further adjusted for history of hypertension, diabetes, cerebrovascular diseases, cancer and heart disease

Of the individual SWB items, most participants reported being satisfied with their life (93.5%), optimistic (95.3%), and conscientious (98.4%). Only a few participants had symptoms of anxiety (4.8%), loneliness (7.9%), and feelings of uselessness (24.6%). Table 3 presents the association between individual SWB items and all-cause mortality. After entering all eight items in model 3, only 2 symptoms (feelings of uselessness: HR = 0.94, 95% CI: 0.89–0.99; and happiness: HR = 0.91, 95% CI: 0.86–0.95) were significantly associated with all-cause mortality.

**Table 3:** Association between specific symptoms and all-cause mortality

Items	Symptoms No. (%)	HR (95%CI)	P value
Life satisfaction	12,420 (93.5)	1.01 (0.92,1.10)	0.843
Optimism	12,655 (95.3)	0.95 (0.86,1.05)	0.331
Conscientiousness	13,066 (98.4)	0.89 (0.75,1.04)	0.150
Anxiety	640 (4.8)	0.91 (0.82,1.01)	0.075
Loneliness	1044 (7.9)	0.96 (0.88,1.04)	0.314
Personal control	11,250 (84.7)	0.97 (0.92,1.03)	0.348
Uselessness	3268 (24.6)	0.94 (0.89,0.99)	**0.020**
Happiness	9027 (68.0)	0.91 (0.86,0.95)	**0.000**

HR: hazard ratio; CI: confidence interval. The model was adjusted for the eight items of individual subjective well-being, age, sex, marital status, educational level, place of residence, smoking, drinking, dietary diversity, exercise and BMI, and history of hypertension, diabetes, cerebrovascular diseases, cancer and heart disease

**Fig. 2:**
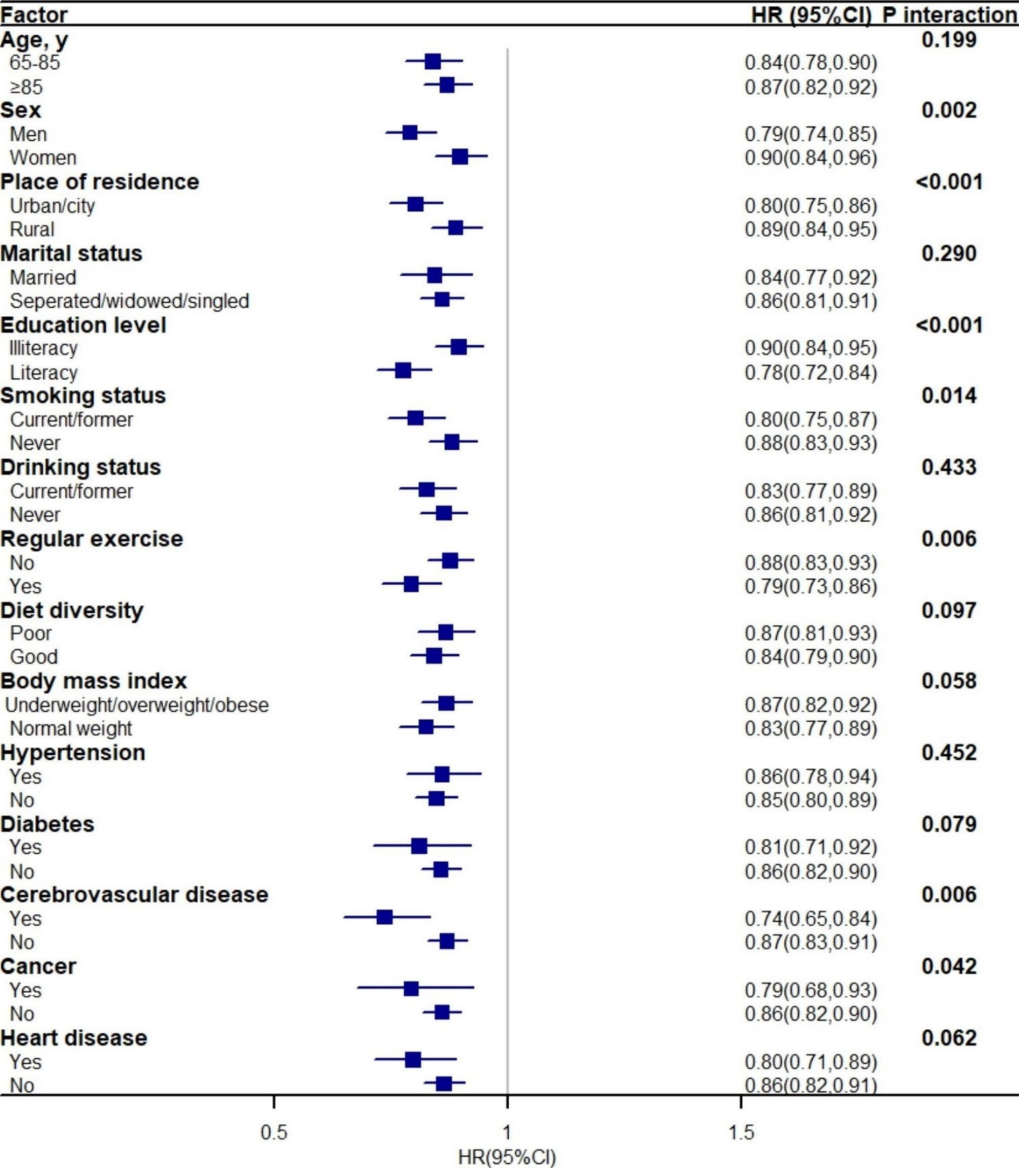
Association between subjective well-being and all-cause mortality stratified by different factors. Graph shows the hazard ratios (HRs) and 95% CIs for all-cause mortality, adjusted for age, sex, marital status, educational level, place of residence, smoking, drinking, dietary diversity, exercise and BMI, and history of hypertension, diabetes, cerebrovascular diseases, cancer and heart disease

Figure 2 presents the association between SWB and all-cause mortality stratified by all potential risk factors. The association between SWB and all-cause mortality remained significant across all subgroups, although it was more evident among males, those living in urban/city settings, literacy, taking regular exercise, and with cerebrovascular diseases (all p < 0.01 for interaction). In addition, several sensitivity analyses were conducted, and the results are shown in the supplementary file. Overall, the relationship between SWB and all-cause mortality was confirmed in these analyses, with the better SWB group having a lower risk of all-cause mortality than those with worse SWB (supplementary Table S1). Moreover, participants who were “unable to answer” for the SWB items experienced the lowest survival over the follow-up duration, and participants with a higher SWB score experienced a lower risk for all-cause mortality (supplementary Fig. 1 and Table S2).

## Discussion

In this nationally representative cohort of 13,282 older adults aged 65 ≥ years with an average follow-up of 16.5 years, we found that a greater level of SWB was significantly associated with a reduced risk of all-cause mortality after adjusting for a wide range of potential confounders. These findings may have both individual and public implications, as the results suggest that a healthier SWB is similarly associated with a lower risk of total mortality regardless of the presence of different baseline characteristics. In addition, the presence of specific SWB items (feelings of uselessness and happiness) was independently associated with the risk of all-cause mortality.

We found that participants with better SWB had a 15% lower risk of all-cause mortality than those with worse SWB in the follow-up duration, which is consistent with previous studies [14, 35]. A number of observational studies also reported a positive association between greater SWB and reduced mortality risk in both the general population and diseased individuals [11, 36]. Despite the existence of publication bias and small size effects, a meta-analysis of 62 cohorts covering 1,250,000 general participants in 2017 supports our results by showing a pooled HR of 0.92 (95% CI = 0.91–0.93) for individuals with higher SWB at baseline in comparison with those with lower SWB, indicating that higher SWB might be a protective factor for total mortality [17]. However, there were a few studies that failed to confirm the association between SWB and survival [11, 15, 37]. The Million Woman Study (MWS) surveyed 719, 671 UK women and found that happiness and related well-being did not have a direct impact on total mortality, with an adjusted rate ratio (RR) of 0.98 (95% CI = 0.94–1.01) [15]. Potential explanations for the variations in results may include the differences in the characteristics of study populations (e.g., age, sex composition, ethnicity) and various measurements of SWB. For instance, the MWS only focused on females aged 55 to 63 years, but findings in our analysis and previous studies suggested that the SWB-survival associations were more pronounced in men than in women [17, 38], and studies of younger individuals may have limited power due to lower event rates (55–63 years in MWS vs. 65 years and above in CLHLS). In addition, at present, there are no perfect or agreed approaches to measure SWB, thus limiting the comparability between studies. SWB in the MWS was assessed by a single question with a four-point scale, while in the current study, a more comprehensive tool comprising eight aspects of SWB, was introduced to measure individual SWB.

Although the association between SWB and mortality has been widely assessed, the contribution of individual SWB items to total mortality after adjusting for each other is still unclear. This study found that self-perception of uselessness and happiness were independently associated with the risk of all-cause mortality after adjustment for all potential confounders and other SWB symptoms. Previous studies have reported a positive association between self-perception of uselessness and an increased risk of mortality [38–40], which is consistent with our findings that participants who had no feelings of uselessness experienced a 6% lower risk of total mortality than their counterparts who had feelings of uselessness. A prospective study of older Italian people found that the feeling of uselessness was associated with an elevated mortality risk at a 3-year follow-up duration [39]. The MacArthur Study of Successful Aging also supports our findings by showing that older adults with feelings of uselessness experienced a greater risk of mortality (adjusted HR = 1.75, 95% CI = 1.22–2.51) during the subsequent 9-year follow-up [40]. The mechanisms linking feelings of uselessness and mortality remain uncertain but may be categorized as psychosocial, behavioral and/or physiological pathways [41–43]. First, feelings of uselessness may diminish one’s self-efficacy, which is important for their social relationships and self-perception of social support [44], and this may lower their capacity to achieve psychosocial well-being. The latter is a potential predictor for mortality [45]. Second, feelings of uselessness may lead to poor health behaviors [46]. For example, older people who feel useless with age may be less likely to seek medical healthcare services (e.g., cholesterol tests) to respond to their deteriorating health [47]. Finally, the self-perception of uselessness may increase the total mortality risk by a variety of complex and interrelated biological pathways, such as the alteration of neuroendocrine hormones, dysfunction of the immune system and central neurotransmitter system as well as the dysregulation of the cardiovascular system [11, 48].

The existing investigations about the association between happiness and all-cause mortality have shown mixed results [49, 50]. The General Social Survey-National Death Index (GSS-NDI) found that the risk of mortality over the follow-up period is 14% higher among participants who are not happy than among those who are very happy [50], which is similar to our findings. However, a few studies reported no significant relationship between happiness and all-cause mortality [49]. The discrepancies may be mainly explained by the various measures of happiness. The happiness-mortality relationship may be explained by many potential mechanisms. On the one hand, happiness is related to lower self-perceived stress, and better immune function, which might protect participants against illness [51, 52]. On the other hand, happy people tend to have better health outcomes because of their greater resilience and higher capacity, and better adaptation abilities to deal with adversities [53].

Although associations between SWB and all-cause mortality are stronger among males, those living in urban/city regions, those with literacy and individuals with cerebrovascular diseases, the SWB-mortality relationship remained significant across all subgroups, highlighting the necessity of promoting SWB among older people regardless of their different characteristics. In addition, our findings also raise an important issue of whether participants with diseases can benefit from greater SWB. Among individuals with hypertension, diabetes, heart disease and/or cancer at baseline, the relationships between SWB and total mortality were similar to those in general populations. The considerable reductions in the risk of all-cause mortality further add to the existing body of literature, indicating that promoting a healthier SWB is meaningful for people with disease [36].

SWB may impact the risk of all-cause mortality through the following two pathways. The first facet is health-related behaviors. A study reported a bidirectional relationship between SWB and heath behaviors, including physical activities, dietary habits, alcohol consumption, and smoking status [54]. Another investigation conducted in a sample of Portuguese adults also found that the SWB level was closely associated with the Mediterranean diet (MD) [55], which may reduce their rates of total and cardiovascular death [56]. Furthermore, SWB may have an influence on total mortality by altering people’s healthcare seeking behaviors [11]. For instance, the analysis of the Health and Retirement Study showed that participants with greater purpose in life were more likely to have cholesterol tests, colonoscopy, and prostate examinations (only men) [47]. Nevertheless, in the current study, we controlled for a list of health behaviors, including regular exercise, dietary diversity, smoking, drinking and BMI, and the SWB-mortality association remained stable. The second facet is through biological process. On the one hand, SWB is related to a variety of metabolic parameters (e.g., plasma cholesterol and glycated hemoglobin) [57, 58]. Previous evidence has shown that people with better SWB have lower levels of cortisol than those with worse SWB, which may help reduce their disease vulnerability, thus lowering the mortality risk [59]. Many investigations have also suggested that SWB may exert its influence by dysregulating of multiple systems (e.g., the immune system, cardiovascular system, and inflammatory reactions). For example, SWB has been found to be related to many inflammatory biomarkers including interleukin(IL)-10, IL-4, and IL-6 [60]. In addition, given the heritability of both SWB and lifespan [61], researchers suspected that some genetic factors may contribute to the association between SWB and survival. However, one study in Danish populations adopted a twin design to deal with the potential confounding effects of genes, and their findings indicated that SWB was associated with mortality independent of genetic factors [62]. More investigations from other populations are needed to address this issue.

### Strengths and limitations

The main strengths of the current study included a large sample size, longitudinal design, large number of accumulated death cases, adjustment of a wide range of confounders, a long follow-up duration, and the conduct of a series of supplementary analyses, which improved the extrapolation validity and robustness of our findings. Additionally, a comprehensive measurement with eight items covering life satisfaction, four positive affect and three negative affect was used to evaluate SWB in our study. This enables us to explore the specific associations of each SWB symptom with mortality, which is above the other measure of SWB. Finally, this study presented an attempt to understand the relationship between SWB and longevity among older people in China, emphasizing the importance of promoting SWB later in life.

However, several limitations should also be noted. First, deaths from specific causes, such as death due to cardiovascular disease and cancer, were not analyzed in our study due to the unavailability of these data in the CLHLS. Second, we excluded approximately 14% of the participants who were unable to answer the question for SWB symptoms. This may lead to a healthier sample, which could underestimate the effect of SWB on total mortality. However, in one of the sensitivity analyses, we incorporated these individuals in our analysis and classified them into a separate group; the results showed that participants who were unable to answer during the survey did have a higher risk of mortality than those included in our analysis. Nevertheless, our study still found a positive SWB-mortality relationship, further highlighting the importance of promoting greater SWB. Third, although we adjusted for a wide range of potential confounders, residual confounding and reverse causality may exist. To address the reverse causality issue, we carried out several sensitivity analyses by excluding participants with life-threatening illness at baseline (those with cancer, heart disease and cerebrovascular diseases), those aged 90 years and above (who are more likely to die during the follow-up), those underweight (BMI < 18.5 kg/m^2^, a signal of poor health for older adults) [63], and those who died within the first year of follow-up. Repeated analyses showed the robustness of our findings. Fourth, chronic physical comorbidities were self-reported, and it is impossible to verify these conditions via medical records since they are unavailable in the CLHLS, which may lead to misclassifications. However, considering that many chronic physical diseases, such as cerebrovascular diseases, cancer and heart disease are serious medical conditions, and patients would generally seek professional medical examinations and treatments, this may not affect our conclusions considerably. In addition, previous studies suggested that self-reported physical conditions (e.g., cerebrovascular diseases and heart disease) have sufficient validity by comparing self-reported physical comorbidities with those identified from multiple information sources, including hospital records [64–66]. Finally, the sample included in the current study only consisted of older Chinese people, and thus, the results should be interpreted conservatively in other populations.

### Implications

China is transforming into an aging nation rapidly in recent decades, and a consequence is that there is a surge in the prevalence and incidence of age-related diseases, such as cancers, chronic noncommunicable diseases, and mental disorders, which present formidable challenges to the healthcare system in China [67]. At the same time, elderly individuals are more likely to have poor SWB due to the loss of family or friends and/or decreased physical or cognitive function. Previous studies have reported a downward trend in SWB levels with the increase of age [68]. In addition, with the development of the economy and urbanization in China, the traditional family structure was eroded, giving rise to the “empty nest” phenomenon, which may impact the SWB level of the elderly due to the lack of family support. For instance, researchers have found that “empty nest” elders are less likely to having higher life satisfaction, and tend to suffer from a higher incidence of depression and loneliness [69]. However, unfortunately, the SWB of the elder people is often neglected in China. It is of great necessity to develop an affordable and comprehensive strategy to promote a healthy and long lifespan for elderly individuals in China. Our findings that the level of SWB, especially the feelings of happiness and uselessness, is significantly associated with the risk of all-cause mortality not only contribute to the limited evidence on the SWB-mortality association but are also important to the scarce research on this association in China, where affordable and effective strategies and programs are necessary to respond to the growing burden of the aging population. The effect of SWB on longevity should also be further confirmed in other large prospective cohort studies and/or intervention studies from different populations. More attention should be given to the subjective dimension of well-being among older people in future research and health programs.

## Conclusions

In conclusion, this study found that better SWB is associated with a reduced risk of total mortality among the older Chinese population, contributing to the limited evidence on this association in LMICs and China. The presence of certain SWB symptoms, including feelings of uselessness and happiness is related to the risk of all-cause mortality. In addition, the findings of this study also suggest that promoting greater SWB could significantly improve longevity regardless of baseline characteristics. Future studies are needed to explore the association between SWB and the risk of all-cause and specific-cause mortality in other LMICs.

### Electronic supplementary material

Below is the link to the electronic supplementary material.

Supplementary Material 1 Figure 1

Supplementary Material 2 Figure 2

## Data Availability

The data support the findings of this study are available from the Chinese Longitudinal Healthy Longevity Survey (CLHLS) group (Chinese Longitudinal Healthy Longevity Survey (CLHLS) – Duke Aging Center), but restrictions apply to the availability of these data, which are used under license for the current study, and so are not publicly available. Data are however available from the authors upon reasonable request and with permission of the CLHLS group (AgingCenter@duke.edu).

## Abbreviations

LMICs: Low- and middle-income countries
SWB: Subjective well-being
CLHLS: Chinese Longitudinal Healthy Longevity Survey
BMI: Body mass index
SDs: Standard deviations
HRs: Hazard ratios
CIs: Confidence intervals
MWS: The Million Woman Study
GSS-NDI: The General Social Survey-National Death Index
MD: Mediterranean diet
