# Supplementary figures and images for: Association between subjective well-being and all-cause mortality among older adults in China
Source: BMC Psychiatry. 2023 Aug 25;23:624. doi: 10.1186/s12888-023-05079-y (PMC10463678; doi:10.1186/s12888-023-05079-y)

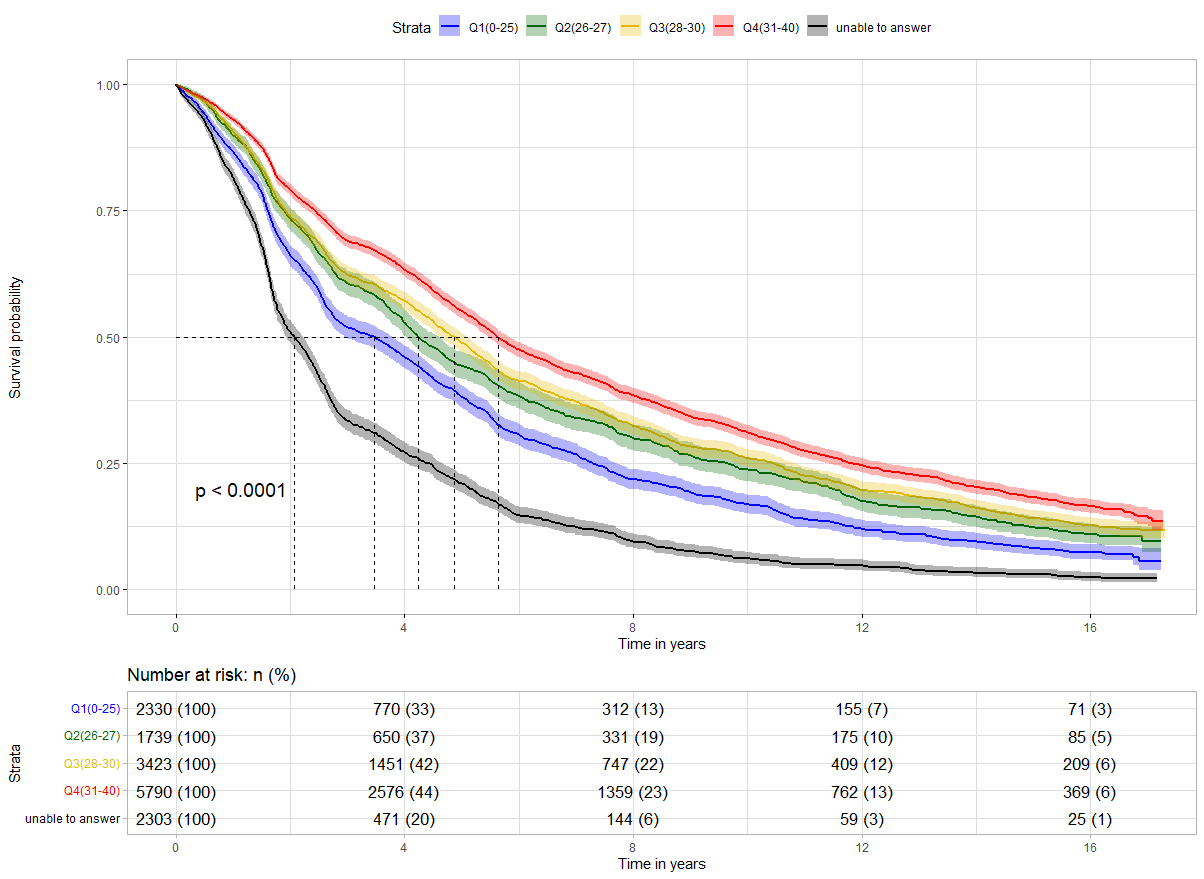

Supplement: Supplementary file 1 — Supplementary Material 1 Figure 1 [file 12888_2023_5079_MOESM1_ESM.tiff]
